# Impact of RSBY on enrolled households: lessons from Gujarat

**DOI:** 10.1186/1753-6561-6-S5-O9

**Published:** 2012-09-28

**Authors:** Tanya Seshadri, Mayur Trivedi, Deepak Saxena, Werner Soors, Bart Criel, Narayanan Devadasan

**Affiliations:** 1 Institute of Public Health, Bangalore, India; 2 Indian Institute of Public Health, Gandhinagar, India; 3 Institute of Tropical Medicine, Antwerp, Belgium

## Introduction

Launched in 2008, the Rashtriya Swasthya Bima Yojana (RSBY) insurance scheme has the mandate of improving access to quality health services of families living below the poverty line (BPL) while providing financial protection against health shocks. Four years following implementation, it is yet to find its presence in all districts of the twenty-eight States in India. Budgetary limitations, the balance between maintaining the claims ratio and sustainability, the contractual relationship between insurance companies and empanelled hospitals, and the weak monitoring system are few challenges that the scheme faces today.

In this paper, we explore implementation of RSBY based on the three dimensions of universal health coverage (UHC) stated in the World Health Report 2010 i.e. “the proportion of the population to be covered” (breadth of coverage), “the range of services to be made available” (depth of coverage) and “the proportion of the total costs to be met” (height of coverage).

## Methods

A cross-sectional study was conducted in Patan district in Gujarat using both qualitative and quantitative methods. A household survey was conducted for 3,120 BPL households (17,420 members). Focus group discussions were conducted among these households separately for those enrolled and those who had not. To understand the context of RSBY in Gujarat and the study findings, in-depth interviews were conducted among other actors from government officials to service providers.

## Results

Limited awareness of the details of the scheme and its benefits was found to be the most important reason for non-enrolment and non-utilisation amongst enrolled members. It was found that 8% enrolled households never received the card while 30% members of cardholding households were not registered on the card making them not eligible for using RSBY. Among non-insured members in enrolled households, a significantly higher prevalence of women was seen. Of the under-fives, 76% remained uninsured.

Among hospitalisations, the inpatient admission rate among insured members was found to be significantly high (39/1,000) along with utilisation rate of 2.2% when compared to the non-insured and non-enrolled. Around two-thirds of insured users underwent surgical treatment and this was 2.8 times higher than those who did not use the scheme. Due to various concerns with medical packages’ reimbursements, many providers have either completely stopped or limit the use of RSBY for non-surgical treatment.

Among the insured users who were hospitalised, only 15% had a cashless experience. The remaining had some out-of-pocket (OOP) expenditure; the median expenditure was found to INR 7,000, similar to those who had not used the scheme or were not enrolled (INR 7,000 and INR 8,000 respectively). This varied greatly between packages. Among the top three packages, 83% of hysterectomy patients, 55% of cataract surgery patients, and 72% of deliveries had some OOP; the median OOP payment to the providers being an additional 100-160% of the actual package rates.

## Discussion

In 2011-12, only 45.3% of the eligible BPL households have enrolled in the scheme as per official RSBY figures. This implies that the scheme currently covers 20% of the general population i.e. the bottom quintile considering the average household size of five (Census 2011). As per the design of the scheme, being enrolled implies being insured. However, this study reveals that even in enrolled households a significant proportion of the population remains non-insured due to various reasons and thus is unable to utlilise the scheme. This reduces the breadth of coverage further. RSBY covers inpatient services at the secondary level of health services only and based on the findings, mainly surgical services. The issues the non-surgical packages needs to be resolved as a priority barring which preference for surgical treatment will rise among providers and beneficiaries. The most significant finding was the near absence of financial protection offered by the scheme and calls for strict monitoring at the level of utilisation.

Addressing the concerns laid out by this study will help the scheme to mature considerably. Questions of sustainability, permanence and expansion to those above poverty line will also need to be addressed if RSBY wants to be considered a tool for achieving UHC in India.

## Competing interests

Authors declare that they have no conflict of interest.

## Funding statement

The study was funded by the Alliance for Health Policy and Systems Research, World Health Organization.

